# Hsa_circ_0001017 promotes cell proliferation, migration and invasion in osteosarcoma by sponging miR-145-5p

**DOI:** 10.1186/s13018-022-03062-z

**Published:** 2022-03-28

**Authors:** Qinglei Yang, Hongying Yu, Konghe Hu

**Affiliations:** 1 Department of Arthropathy and Osteopathy, Yuebei People’s Hospital Affiliated to Shantou University Medical College, No.133 Huimin South Road, Wujiang District, Shaoguan City, 512000 Guangdong Province People’s Republic of China; 2 Department of Pharmacy, Yuebei People’s Hospital Affiliated to Shantou University Medical College, Shaoguan, 512000 China

**Keywords:** Osteosarcoma, Circular RNA microarray, circ_0001017, miR-145-5p

## Abstract

**Background:**

Circular RNAs (circRNAs) have displayed important roles in the development and progression of various cancers. However, the functions of the majority of circRNAs in osteosarcoma (OS) remain unknown.

**Methods:**

Circular RNA microarray analysis was performed in three OS cell lines (Saos-2, U2OS and MG63) and normal vascular endothelial cells. The co-differentially expressed circRNAs (CDECs) were identified in OS cell lines with the criterion of FDR (false discovery rate) < 0.05 and |fold change (FC)|> 2. Quantitative real-time PCR was used to validate the expression levels of selected CDECs. A series of functional assays, including MTT assay, flow cytometry and transwell assay were conducted in OS cells. The interaction between circRNA and miRNAs was confirmed by luciferase reporter assay and RNA immunoprecipitation assay.

**Results:**

A total of 241 CDECs, including 75 upregulated and 166 downregulated CDECs, were identified in three OS cell lines compared with normal vascular endothelial cells. PCR validation showed that hsa_circ_0000704, hsa_circ_0001017 and hsa_circ_0005035 were all highly expression in the three OS cell lines, compared with osteoblast cell lines (HECC, hFOB1.19 and HFF-1). Functionally, overexpression of circ_0001017 significantly promoted the cell proliferation, migration and invasion and decreased apoptosis in U2OS cells. Knockdown of circ_0001017 obtained the opposite results. Circ_0001017 may downregulate miR-145-5p through direct binding. Furthermore, the expression of miR-145-5p was negatively regulated by circ_0001017 in OS cells. In addition, further functional studies indicated that miR-145-5p inhibitor eliminated the effects caused by si-circ_0001017 in OS cells.

**Conclusions:**

In conclusion, our study suggested that circ_0001017 may be a novel oncogenic factor during the progression and development of OS by targeting miR-145-5p.

**Supplementary Information:**

The online version contains supplementary material available at 10.1186/s13018-022-03062-z.

## Introduction

Osteosarcoma (OS), derived from the metaphysis of long bones, is a class of primary malignant bone tumor frequently occurred in children and young adults [1]. OS is clinically characterized as abnormal growth of bone-related mesenchymal cells, formation of osteoid tissue and highly aggressive phenotype [2]. Despite advancement strategies, including wide local tumor excision, adjuvant chemotherapy and radiotherapy, the five-year survival rate of OS patients, especially metastatic or recurrent cases, remains below 30% [3, 4]. Therefore, exploring the molecular mechanism underlying OS initiation and progression is crucial to improve the outcomes of this disease.

Circular RNAs (circRNAs), a class of endogenous non-coding RNAs with covalent and closed-loop structures [5], could function as miRNA molecular sponges to decrease miRNA activity by binding to miRNAs [6]. Increasing evidence manifested that circRNAs exert crucial roles in the biological function and pathogenic mechanism of OS [7]. For instance, silencing hsa_circRNA_0008035 exerted repressive function on OS cell growth and migration and Notch pathway by accelerating miR-375 [8]. Hsa_circ_0003732 could elevate CCNA2 expression via miR-545, resulting in the promotion of OS cells proliferation [9]. In addition, the tumor suppressive effects of hsa_circ_0000658 [10], has_circ_0088212 [11] and hsa_circ_0046264 were also reported in OS [12]. Nevertheless, there findings on the functional role of circRNAs in OS are far from enough. Currently, the advancement of high-throughput microarray platforms now provides possibility for us to perform comprehensive and systemic analysis of circRNAs profiling analysis in OS.

In the present study, we first performed circular RNA microarray analysis in three OS cell lines and identified differentially expressed circRNAs (DECs) between OS cell lines and vascular endothelial cells. After validating the DECs, we perform a series of functional experiments to investigate the role of certain circRNA in OS in vitro. Furthermore, we explored the underling mechanisms underlying these functional roles. Our research might provide the novel therapeutic targets for OS.

## Materials and methods

### Cell culture

The human OS cell lines (Saos-2, U2OS and MG63) and osteoblast cell lines (HECC, hFOB1.19 and HFF-1) were purchased from the American Type Culture Collection (USA). All cell lines were cultured in Dulbecco's modified Eagle medium (cat. No. 11320033; Gibco; Thermo Fisher Scientific Inc., USA) with 10% fetal bovine serum (cat. No. 16140071; Gibco) in a cell incubator with 5% CO_2_ at 37 °C.

### Circular RNA microarray analysis

Total RNA was extracted from three OS cell lines (Saos-2, U2OS and MG63) and normal vascular endothelial cells, of which linear RNA was removed by RNAse R kit (Epicentre, Inc., Madison, WI, USA). Then, the enriched circRNAs were amplified and transcribed into fluorescent cRNAs. The labeled cRNAs were hybridized onto the Arraystar Human circRNA Array (8 × 15 K; Arraystar). Next, the arrays were scanned by the Agilent scanner G2505C, and the obtained array images were analyzed using Agilent Feature Extraction software (11.0.0.1 version). Afterward, the R software limma package was used to perform quantile normalization and subsequent data processing. Differentially expressed circRNAs (DECs) between OS cell lines and vascular endothelial cells were identified through Volcano plot filtering with the inclusion criteria of FDR (false discovery rate) < 0.05 and |fold change (FC)|> 2. The overlapping DECs in three OS cell lines *vs.* vascular endothelial cells were selected as co-differentially expressed circRNAs (CDECs).

### Quantitative real‐time PCR

Total RNA from cell lines was isolated using TRIzol reagent (Invitrogen), and cDNA was synthesized with a One Step PrimeScript® circRNA cDNA Synthesis Kit (Takara, Shiga, Japan) in accordance with the reagent instructions. Quantitative real-time analysis was conducted on the ABI Prism 7500 Detection System (Applied Biosystems, USA) using the SYBR Premix Ex Taq kit (Takara, Shiga, Japan). Relative gene expression levels were calculated according to the 2^−ΔΔCT^ method with GAPDH or U6 as the internal control.

### Cell transfection

Human circ_0001017-overexpressing vector (circ_0001017), the control vector plasmid, small interfering RNA targeting circ_0001017 (si-circ_0001017) and negative control (si-NC) were synthesized by GeneCopoeia (Rockville, MD, USA). miR-145-5p mimics, inhibitor and miR-NC were purchased from Genema (Shanghai, China). For cell transfection, U2OS cells were transfected with the above-mentioned oligonucleotides by the Lipofectamine® 3000 transfection reagent (Invitrogen). After 48 h of transfection, cells were collected for further analysis.

### MTT assay

MTT assay was performed for analysis of cell proliferation. In brief, cells from different groups were seeded into 96-well plates at a density of 3,000 cells per well and cultured for 0, 24, 48 and 72 h. At each time point, cells in each well were incubated with 10 μL MTT solution (0.5 mg/mL, Beyotime) for 2 h at 37 °C. Subsequently, 100 μL acidic isopropanol (10% SDS, 5% isopropanol and 0.01 M HCl) was added into the medium to stop the reaction. The optical density (OD) values at 595 nm were determined using a microplate reader (BIO-TEK, Winooski, VT, USA).

### Cell apoptosis analysis

In brief, cells from different groups were harvested and washed twice with ice-cold PBS. After adjusting a concentration of 1 × 10^6^ cells/mL through re-suspension in 1 × binding buffer, cells were incubated with 5 µl 1 × Annexin V/FITC and 10 µl propidium iodide (PI) for 15 min at room temperature in dark. Next, the percentage of apoptotic cells was analyzed using flow cytometry (FACSCalibur, BD, Franklin Lakes, NJ, USA).

### Transwell assay

For cell migration assay, approximately 5 × 10^4^ cells in 200 µl of serum‐free medium were seeded in the upper transwell chambers (8 μm pore size, Corning, MSA, USA) and 500 µl of medium containing 10% FBS was added to the lower chamber. After 24-h incubation at 37 °C, the cells that migrated to the lower chamber were fixed with 95% ethanol for 15 min, stained with 0.1% crystal violet for 20 min and counted across five randomly selected fields under a microscope. The procedures of cell invasion assay were the same as the migration assay, except for the upper chamber coated with diluted Matrigel (BD Biosciences, San Diego, CA, USA).

### Luciferase reporter assay

The potential binding sites of miR-145-5p and circ_0001017 were obtained from StarBase v3.0 (http://starbase.sysu.edu.cn/). For luciferase reporter assay, the wild-type (WT-circ_0001017) and mutant luciferase reporter plasmids (MUT-circ_0001017) were generated through inserting the wild-type and mutant-type sequences of circ_0001017 containing miR-145-5p complementary sites within pmirGLO (Promega, Madison, WI, USA), respectively. U2OS cells (4 × 10^4^ cells/well) were cultured in 24-well plates overnight and transfected with 400 ng of WT-circ_0001017 or MUT-circ_0001017, together with 50 nM miR-145-5p mimics or miR-NC using Lipofectamine® 3000 transfection reagent (Invitrogen). After incubation for 48 h, relative luciferase activity was measured using a Dual Luciferase Assay Kit (Promega, Madison, WI, USA).

### RNA immunoprecipitation (RIP) assay

RIP assay was performed with Magna RIP RNA-Binding Protein Immunoprecipitation Kit (Millipore, Billerica, MA, USA) according to the manufacturer’s instructions. Briefly, cells were lysed with RIPA lysis buffer and the obtained cell lysates were 5 μg of magnetic beads-coupled anti-Ago2 or anti-IgG antibody (Beyotime, Shanghai, China) at 4 °C overnight. After incubating with proteinase K buffer, we isolated immunoprecipitated RNA with RNasy MineElute Cleanup Kit (Genescript Co, Nanjing, China). The purified RNA was subjected to quantitative real-time PCR to detect the presence of circ_0001017 and miR-145-5p with total RNA as the input control.

### Statistical analysis

All quantitative data were analyzed using GraphPad Prism 6.0 (GraphPad, La Jolla, CA, USA) and expressed as mean ± standard deviation (SD). Differences between two groups were compared using Student’s t test. One-way analysis of variance followed by Tukey’s post hoc test was used for multiple comparisons. A *p* < 0.05 was considered statistically significant.

## Results

### CircRNA microarray analysis revealed the expression profile of circRNA in OS

**Fig. 1:**
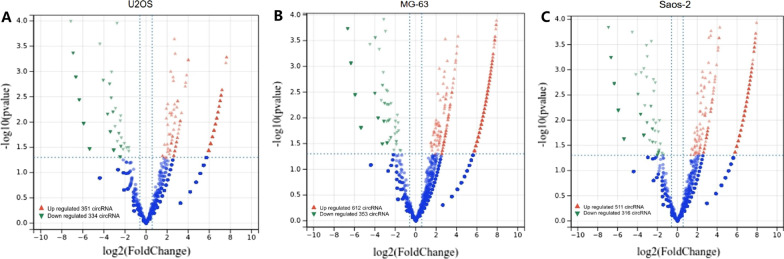
Overview of the microarray signatures. Volcano plot visualizing the differentially expressed circRNAs between U2OS (**A**), MG63 (**B**) or Saos-2 (**C**) cells and normal vascular endothelial cells. The red dots represent upregulated circRNAs and green dots represent downregulated circRNAs based on the filter criteria (fold change ≥ 2.0 and FDR < 0.05)

**Fig. 2:**
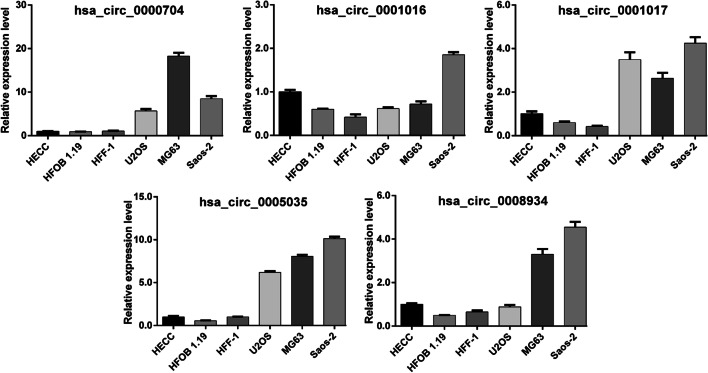
Validation of five circRNAs. Quantitative real-time PCR was used to determine the expression levels of hsa_circ_0000704, hsa_circ_0001016, hsa_circ_0001017, hsa_circ_0005035 and hsa_circ_0008934 in human OS cell lines (Saos-2, U2OS and MG63) and osteoblast cell lines (HECC, hFOB1.19 and HFF-1)

To investigate the abnormal expression profile of circRNAs in OS, we analyzed three OS cell lines with normal vascular endothelial cells using the circRNA microarray. Results revealed that there were a total of 685 abnormally expressed circRNAs in U2OS cells (Fig. 1A), 965 abnormally expressed circRNAs in MG63 cells (Fig. 1B) and 827 abnormally expressed circRNAs in Saos-2 cells (Fig. 1C), compared with normal vascular endothelial cells. After further screening, a total of 241 CDECs were commonly abnormally expressed in three OS cell lines, of which 75 were upregulated and 166 were downregulated in OS cells. Subsequently, we selected several circRNAs, associated with tumorigenesis to examine their expression levels in OS cell lines using quantitative real-time PCR. Among them, hsa_circ_0000704, hsa_circ_0001017 and hsa_circ_0005035 were all highly expression in the three OS cell lines, compared with osteoblast cell lines (HECC, hFOB1.19 and HFF-1) (Fig. 2), which was consistent with the results of our microarray analysis. Combined with related reports, hsa_circ_0001017 was selected for subsequent experiments.

### *Circ_0001017 enhances the proliferation, migration and invasion of OS cells *in vitro

**Fig. 3:**
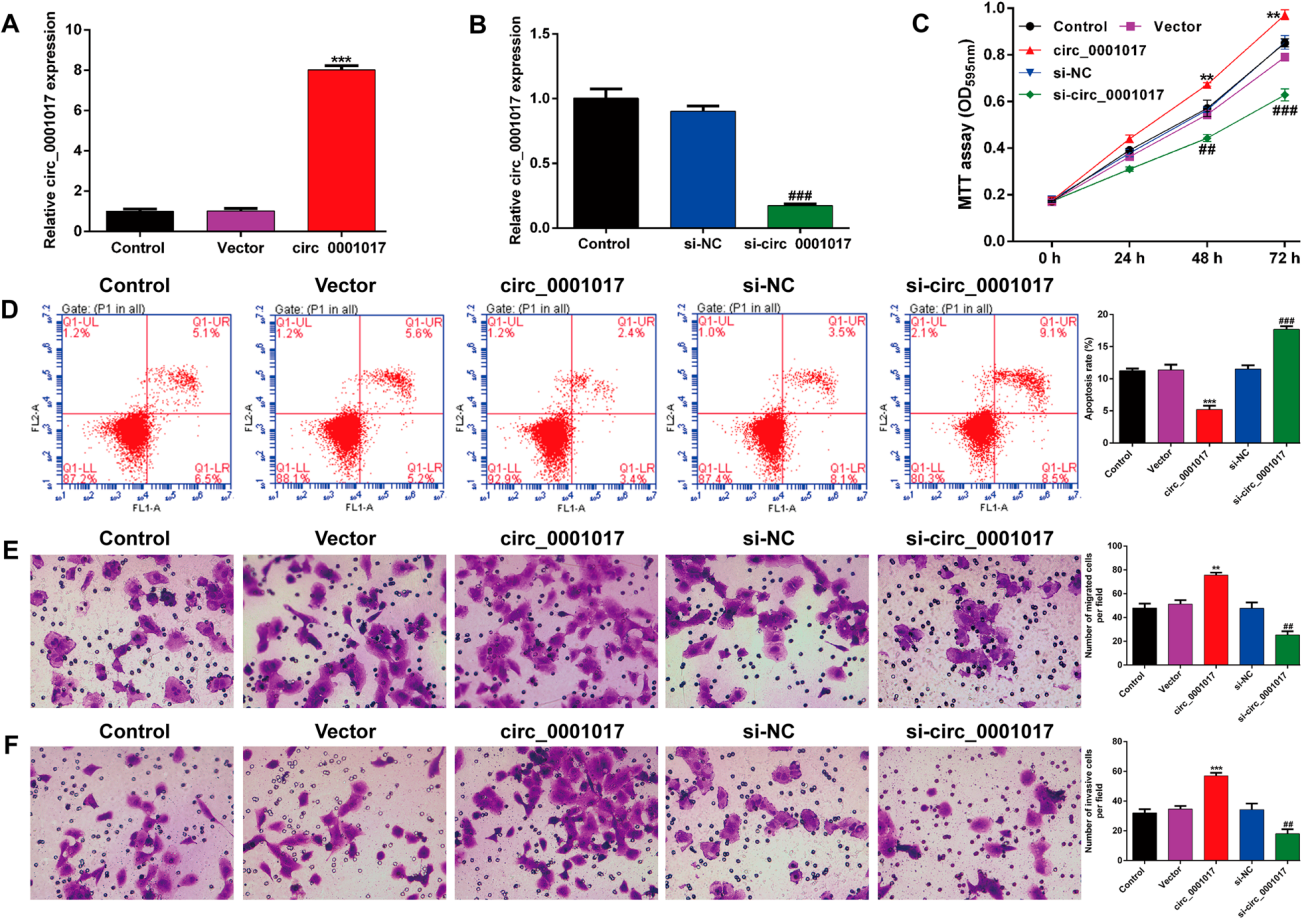
Effects of circ_0001017 on the proliferation, migration and invasion of OS cells. Quantitative real-time PCR was utilized to analyze the expression of circ_0001017 in U2OS cells after transfection with circ_0001017 overexpression plasmid (**A**) or si-circ_0001017 (**B**). **C** MTT assay was used to assess cell proliferation of transfected U2OS cells. (D) Cell apoptosis of U2OS cells was determined by flow cytometry. **E**, **F** Transwell assay was applied to determine the cell migration and invasion capacities. Morphologic changes were examined under a fluorescence microscope at 200 × magnification. **p < 0.01, ***p < 0.001, compared with Vector; ##*p* < 0.01, ###*p* < 0.001, compared with si-NC

To explore the functional role of circ_0001017 in OS cells, three OS cell lines (Saos-2, U2OS and MG63) were transfected with circ_0001017 overexpression plasmid, empty vector, si-circ_0001017 or si-NC. Among the three OS cell lines, only U2OS cells presented higher transfection efficiency compared with the other two OS cell lines, which were thus screened as the cell model of performing gain-of-function and loss-of-function assays. First, quantitative real-time PCR showed that the expression of circ_0001017 was significantly increased after circ_0001017 overexpression plasmid transfection (Fig. 3A), but decreased after si-circ_0001017 transfection (Fig. 3B) in U2OS cells. Subsequent MTT assay revealed that overexpression of circ_0001017 significantly promoted the cell proliferation, while circ_0001017 knockdown suppressed the cell proliferation in U2OS cells (Fig. 3C). Consistent with the cell growth trend, the cell apoptotic rate was obviously decreased after circ_0001017 overexpression, but increased after circ_0001017 knockdown in U2OS cells (Fig. 3D). In addition, transwell assay indicated that the number of migrated (Fig. 3E) and invasive (Fig. 3F) cells was remarkedly elevated following circ_0001017 transfection, but reduced following si-circ_0001017 transfection in U2O cells.

### Circ_0001017 sponged miR-145-5p

**Fig. 4:**
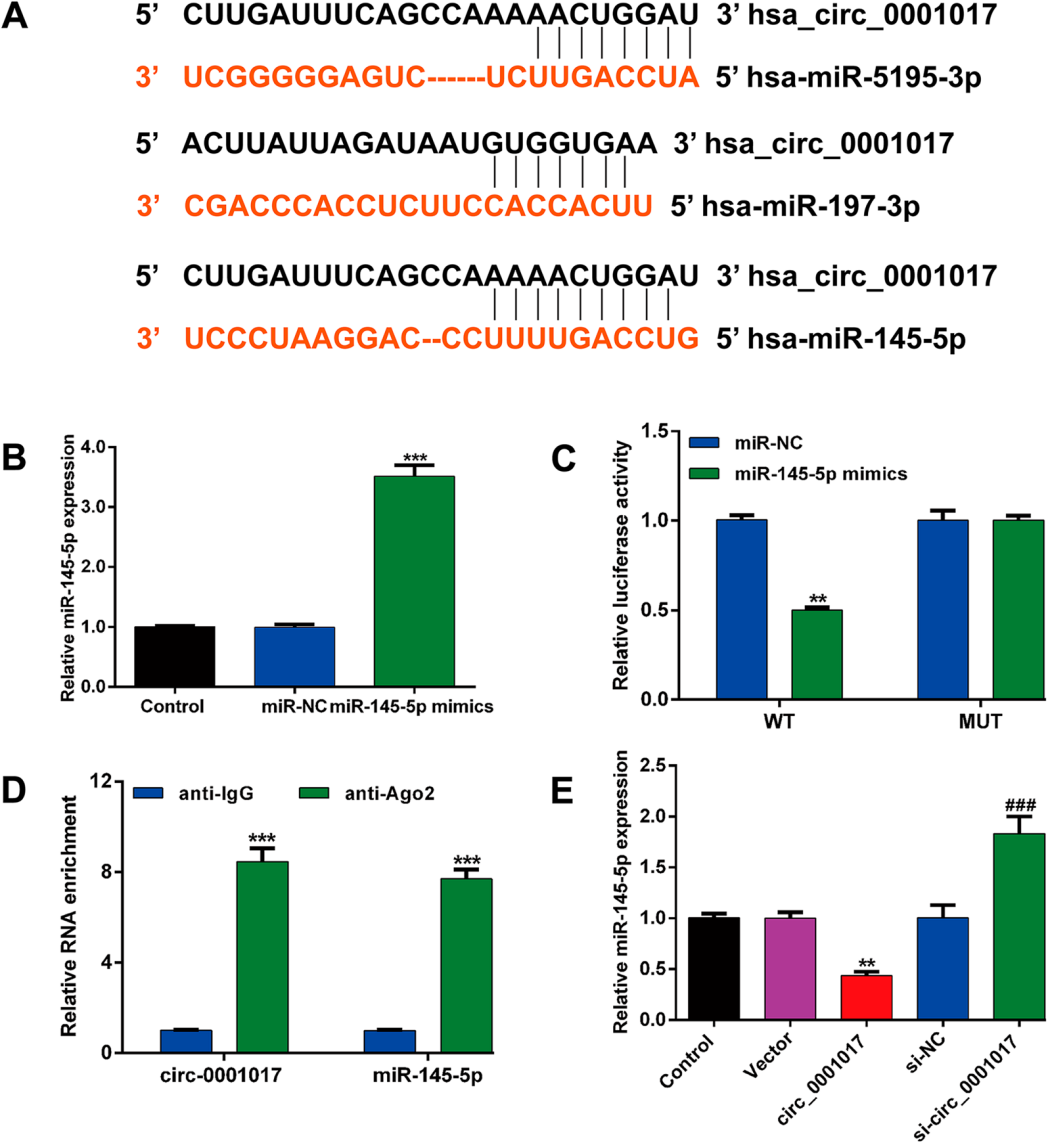
Circ_0001017 sponged miR-145-5p. **A** Schematic presentation of the putative binding sites of three miRNAs with respect to circ_0001017. **B** Quantitative real-time PCR was used to determine the expression level of miR-145-5p in U2OS cells after transfection with miR-145-5p mimics. **C** Luciferase reporter assay in U2OS cells co-transfected with miR-145-5p mimics or miR-NC and reporter constructs carrying wild-type or mutant miR-145-5p binding sequence of 3′-UTR of circ_0001017. **p < 0.01, ***p < 0.001, compared with miR-NC. **D** RIP assay was conducted to examine the enrichment of miR-145-5p and circ_0001017 in the Ago2 complex. ***p < 0.001, compared with anti-IgG. **E **Quantitative real-time PCR was performed to determine miR-145-5p levels in U2OS cells in response to circ_0001017 overexpression or knockdown. **p < 0.01, compared with Vector; ###*p* < 0.001, compared with si-NC

We then performed StarBase v3.0 software analysis and identified three miRNAs (miR-5195-3p, miR-197-3p and miR-145-5p) as potential downstream targets of circ_0001017 (Fig. 4A). Considering that miR-145-5p was previously reported to be downregulated in OS [13], we thus selected miR-145-5p as a potential target of circ_0001017 to further analysis. After confirming the overexpression of miR-145-5p (Fig. 4B), we performed luciferase reporter assay to validate the association between circ_0001017 and miR-145-5p. The results showed that miR-145-5p mimics significantly inhibited the luciferase activities of the WT reporter but not the MUT reporter (Fig. 4C). Moreover, the RIP assay showed that both circ_0001017 and miR-145-5p were enriched in the precipitated Ago2 complex (Fig. 4D). These results suggest that circ_0001017 physically interacts with miR-145-5p. We further explored whether circ_0001017 regulates miR-145-5p expression in U2OS cells. As shown in Fig. 4E, circ_0001017 overexpression inhibited miR-145-5p expression, whereas circ_0001017 silencing dramatically enhanced miR-145-5p expression, compared with corresponding controls. Collectively, these results suggest that circ_0001017 sponged miR-145-5p and negatively regulated it in OS cells.

### Downregulation of miR-145-5p reversed the suppressive effects of circ_0001017 knockdown on cell proliferation, migration and invasion in OS cells

**Fig. 5:**
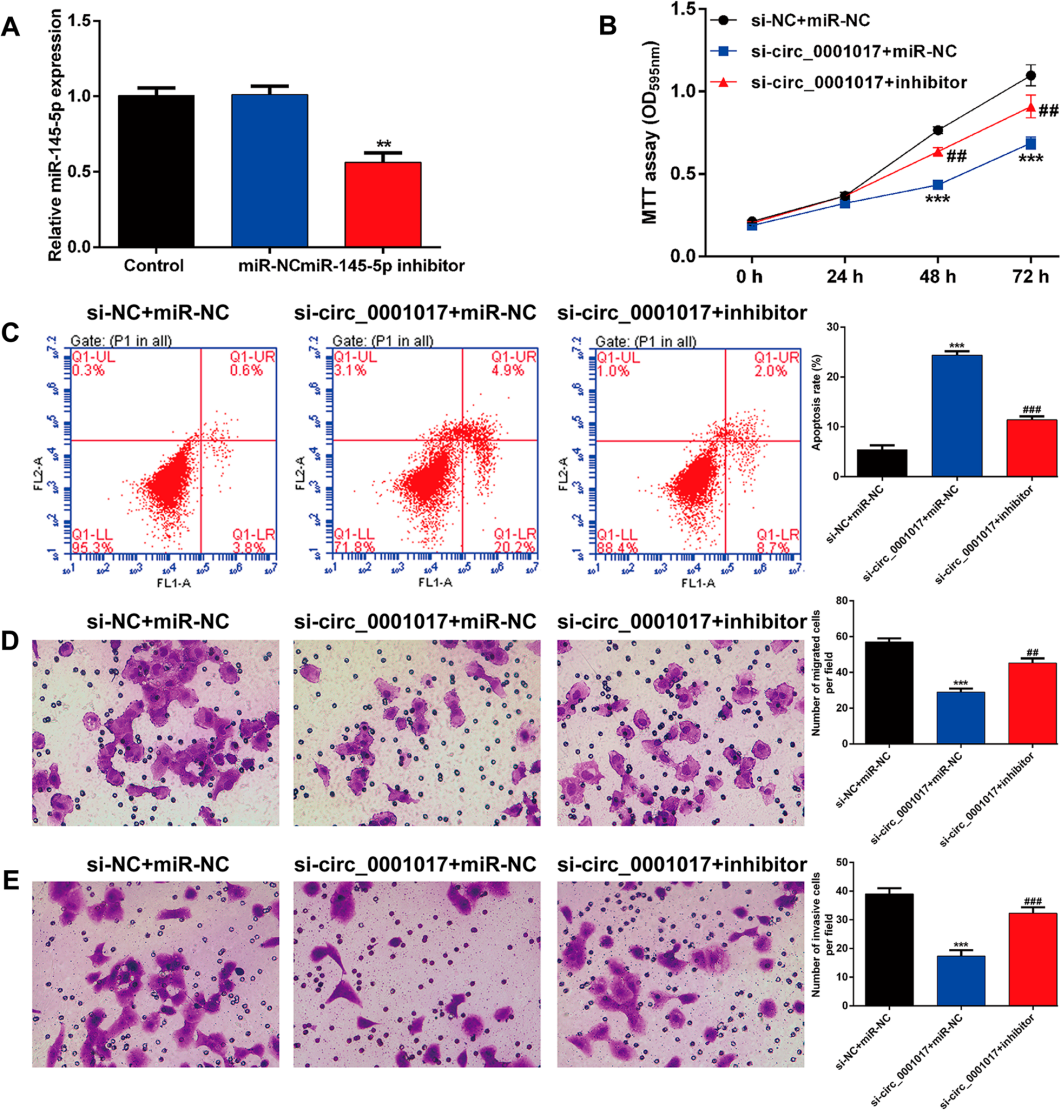
Downregulation of miR-145-5p reversed the suppressive effects of circ_0001017 knockdown on cell proliferation, migration and invasion in OS cells. **A** Quantitative real-time PCR was used to determine the expression level of miR-145-5p in U2OS cells after transfection with miR-145-5p inhibitor or miR-NC. **p < 0.01, compared with miR-NC; **B** MTT assay, **C** flow cytometry and **D**, **E** transwell assay were performed to evaluate cell proliferation, apoptosis, migration and invasion in U2OS cells after co-transfection with miR-145-5p inhibitor and si-circ_0001017. ***p < 0.001, compared with si-NC + miR-NC; ##*p* < 0.01, ###*p* < 0.001, compared with si-circ_0001017 + miR-NC

To explore whether circ_0001017 regulated malignant cellular behaviors in OS by interacting with miR-145-5p, functional rescue experiments were performed in U2OS cells. First, downregulation of miR-145-5p was confirmed using quantitative real-time PCR in U2OS cells after transfection with miR-145-5p inhibitor (Fig. 5A). The results from MTT assay indicated that the miR-145-5p inhibitor dramatically restored the proliferative abilities of circ_0001017-silenced U2OS cells (Fig. 5B). Consistently, flow cytometry analysis demonstrated that miR-145-5p inhibitor dramatically abolished the promoting effects of circ_0001017 knockdown on U2OS cell apoptosis (Fig. 5C). Additionally, downregulation of miR-145-5p partially reversed the decreased migrated (Fig. 5D) and invasive (Fig. 5E) cells induced by circ_0001017 knockdown in U2OS cells. Taken together, these data suggested that circ_0001017 regulated the malignant behavior of OS cells via sponging miR-145-5p.

## Discussion

Abnormally expressed circRNAs have attracted widespread attention in tumor research with the development of high-throughput sequencing technology [14]. CircRNAs have been widely reported to regulate gene expression by serving as competing endogenous RNAs [15]. Moreover, circRNAs serve as key factors in tumor development and progression by regulating a variety of biological processes, including cell proliferation, apoptosis and metastasis [16]. The present study first performed microarray analysis to identify CDECs in OS cell lines. The results identified a total of 241 CDECs, including 75 upregulated and 166 downregulated CDECs in three OS cell lines. Then, we validated the expression levels of five circRNAs, including hsa_circ_0000704, hsa_circ_0001016, hsa_circ_0001017, hsa_circ_0005035 and hsa_circ_0008934 in OS cell lines. Our data showed that hsa_circ_0000704, hsa_circ_0001017 and hsa_circ_0005035 were all highly expression in the three OS cell lines, compared with osteoblast cell lines (HECC, hFOB1.19 and HFF-1). As our best knowledge, hsa_circ_0005035 was notably lower in lung cancer tissues and lung cancer cell lines than in the adjacent normal tissues and cells [17]. Hsa_circ_0001017 expression was decreased in glioma [18] and gastric cancer [19] tissues and cells. The opposite expression levels of hsa_circ_0001017 and hsa_circ_0005035 might be ascribed to different tumor backgrounds.

Subsequently, we selected hsa_circ_0001017 to perform functional assays in OS cell lines. In three OS cell lines, U2OS cells were successfully constructed as hsa_circ_0001017 overexpression and knockdown cell models. Our data indicated that overexpression of circ_0001017 significantly promoted the cell proliferation, migration and invasion and decreased apoptosis in U2OS cells. Knockdown of circ_0001017 obtained the opposite results. These data suggested that circ_0001017 might be an oncogene in the development of OS. Although the oncogenic role of circ_0001017 has not been reported yet, circ_0001017 as a tumor suppressor has been demonstrated as follows: Upregulation of hsa_circ_0001017 could notably muffle the proliferation as well as the metastasis of gastric cancer (GC) cell lines and impede the formation of GC tumor [19]. Overexpression of hsa_circ_0001017 inhibited glioma cell proliferation, migration and invasion and promoted glioma cell apoptosis, while the knockdown of hsa_circ_0001017 caused the opposite results [18]. In addition, enforced expression of circ_0001017 suppressed malignant behaviors and enhanced resistance to cisplatin (CDDP) sensitivity of CDDP-resistant GC cells [20]. These controversial roles of hsa_circ_0001017 in tumors might be mainly ascribed to different tumor resources and types of cell lines.

Moreover, the bioinformatics analysis was performed to anticipate the target miRNAs of hsa_circ_0001017, revealing the binding sites for miR-5195-3p, miR-197-3p and miR-145-5p. In fact, the role of non-coding RNAs in musculoskeletal conditions has been widely reported, including microRNAs (miRNAs) [21, 22] and small interfering RNAs (siRNAs) [23]. According to the report by Wang et al. [24], miR-5195-3p overexpression significantly attenuated OS cell proliferative activity and induced apoptosis. Circ-0003998 upregulates the expression of Krüppel-like factor 10 (KLF10) by binding to miR-197-3p, thereby promoting OS cell growth and invasion [25]. Considering the suppressive effects of miR-145-5p have been reported in OS, we here selected miR-145-5p as a candidate for subsequent research. Further rescue experiments manifested that hsa_circ_0001017 could directly interact with miR-145-5p and miR-145-5p was a functional target of hsa_circ_0001017 in OS. Similarly, circ_0008932 may be a novel oncogenic factor during the progression and development of OS by targeting miR-145-5p [26]. Hsa_circ_0000073 facilitated the proliferation, migration and invasion of OS cells through the inhibition of miR-145-5p [27]. CircCAMSAP1 directly sequesters miR-145-5p in the cytoplasm and inhibits its activity to suppress OS tumorigenesis [28]. Additionally, elevated hsa_circ_0008934 expression promotes the proliferation and migration of OS cells by sponging miR-145-5p [29].

## Conclusions

In conclusion, this study revealed that circ_0001017 is overexpressed in OS cells and that ectopic expression of circ_0001017 promotes cell proliferation, migration and invasion by sponging miR-145-5p (Additional file 1: Figure S1). Our research proposes a novel insight into the pathology of OS and provides a novel target for OS treatment.

## Supplementary Information

**Additional file 1. Figure S1: **The diagram of the mechanism about this research is shown.

## Data Availability

The datasets generated during and/or analyzed during the current study are available from the corresponding author on reasonable request.
