# Supplementary figures and images for: Hsa_circ_0001017 promotes cell proliferation, migration and invasion in osteosarcoma by sponging miR-145-5p
Source: J Orthop Surg Res. 2022 Mar 28;17:184. doi: 10.1186/s13018-022-03062-z (PMC8962139; doi:10.1186/s13018-022-03062-z)

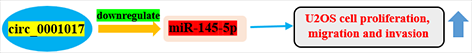

Supplement: Supplementary file 1 — Additional file 1. Figure S1: The diagram of the mechanism about this research is shown. [file 13018_2022_3062_MOESM1_ESM.tif]
